# Growth and Physiological Traits of Blueberry Seedlings in Response to Different Nitrogen Forms

**DOI:** 10.3390/plants14101444

**Published:** 2025-05-12

**Authors:** Haiyan Yang, Yaqiong Wu, Chunhong Zhang, Lianfei Lyu, Wenlong Wu, Zhengjin Huang, Weilin Li

**Affiliations:** 1 Jiangsu Key Laboratory for Conservation and Utilization of Plant Resources, Institute of Botany, Jiangsu Province and Chinese Academy of Sciences (Nanjing Botanical Garden Mem. Sun Yat-Sen), Nanjing 210014, China; haiyanyang@cnbg.net (H.Y.); chzhang@cnbg.net (C.Z.); njbglq@163.com (L.L.); 1964wwl@163.com (W.W.); hzj90@cnbg.net (Z.H.); 2 State Key Laboratory of Tree Genetics and Breeding, Co-Innovation Center for Sustainable Forestry in Southern China, College of Forestry and Grassland, Nanjing Forestry University, Nanjing 210037, China

**Keywords:** blueberry, ammonium nitrogen, nitrate nitrogen, amide nitrogen, amino acids, physiological indexes

## Abstract

This study aimed to better understand the impacts of various nitrogen (N) forms on blueberry growth and development, as well as to increase blueberry (*Vaccinium* spp.) N utilization efficiency. We selected the blueberry cultivar ‘Anna’ as the experimental material, and four N treatments were applied throughout the key vegetative growth stage: N deficiency (CK), ammonium-N (T1), nitrate-N (T2), and amide-N (T3). The growth parameters, physiology indexes, and ultrastructure changes in blueberry seedlings were explored. At the same time, the Pearson correlation model was used to analyze the correlation among each physiology index. The results showed that blueberry plants grew better under T1 and T3 treatments, with increased biomass, N content, chlorophyll content, and photosynthetic efficiency. Under T1 treatment, the leaves had lower O_2_^˙−^ generation rate and MDA concentration, but higher superoxide dismutase (SOD), glutamate synthetase (GOGAT), and glutamine synthetase (GS) activity. Compared to T1 treatment, T2 treatment dramatically enhanced peroxidase (POD) activity, glucose content, and free amino acid content, particularly Arg content. Furthermore, N deficit treatment inhibited plant growth while increasing free radicals, POD, catalase (CAT), and glutamate dehydrogenase (GDH) activities, as well as the content of antioxidant compounds. Correlation and principal component analysis showed that photosynthetic properties, chlorophyll content, antioxidant system, amino acid levels, and N metabolizing enzyme activity were significantly affected by different N forms. This study can serve as a scientific foundation for optimal N regulation and management in blueberries.

## 1. Introduction

Blueberry (*Vaccinium* spp.), a globally important economic small berry fruit tree, offers berries with demonstrated benefits [1], driving increasing demand for both fresh and processed markets [2].

Nitrogen (N), one of the macroelements, is known as the “life element” for the growth of fruit trees [3]. N absorption, distribution, and utilization are strongly related to the growth and development of fruit trees, as well as their yield and quality. N is an essential component of chlorophyll, nucleotides, proteins, as well as various plant hormones, vitamins, and alkaloids, all of which require its presence for their synthesis [4]. To achieve high crop yields, a considerable amount of N fertilizer is applied to increase plant growth and productivity [4,5]. However, blind fertilization will not boost crop production or quality but will raise agricultural expenses, waste fertilizer supplies, and pollute the surrounding environment [5]. It is clear that increasing the N fertilizer-use efficiency in blueberries is critical for promoting the sustainable and healthy development of the blueberry industry.

In agricultural production, N fertilizer can be primarily classified into three forms according to the N-containing group: ammonium (NH_4_^+^), nitrate (NO_3_^−^), and amide-N (urea) [6]. Urea is an organic N source that plants can easily absorb and utilize [7]. As a key component in plant N metabolism, it constitutes the predominant N fertilizer worldwide, accounting for over half of total N fertilizer usage [7]. However, urea must first be degraded into ammonium carbonate by the enzyme urease before being converted into ammonium bicarbonate and ammonium hydroxide, which plants may assimilate and utilize [7]. Ammonium-N (NH_4_^+^) and nitrate-N (NO_3_^−^) are the two primary N types that plant roots take and use from the soil [8]. NH_4_^+^ is absorbed by plants and directly involved in N metabolism with ammonium transporter (AMT). NO_3_^−^ is first transported into a plant by nitrate transporter (NRT), then reduced to NH_4_^+^ under the action of nitrate reductase (NR) and nitrite reductase (NIR), and then assimilated by plants [9].

Due to the differences in the absorption, transport, and assimilation of the three forms of N, different plants exhibit preferences for specific N forms [6]. N form has varying effects on plant growth and development. Therefore, selecting suitable N fertilizer types and application methods is crucial to enhance N-use efficiency and maximize yield benefits in blueberries. Previous research indicated that providing NH_4_^+^ to nitrophilic plants leads to ammonium toxicity [6]. However, in ammoniophilic plants, the NO_3_^−^ absorption system of their roots is reduced [10]. Blueberry is a shallow-rooted plant with a root depth of 12–18 inches, a thin distribution, and no root hairs [11]. Thus, soil fertility and N application are the most important factors influencing blueberry growth and output [8]. Blueberries are particularly sensitive to fertilization, and excessive or insufficient fertilization can stymie growth [9]. Although it has been established that blueberries are ammoniophilic plants, most research focuses on cultivation techniques, such as fertilizer type, application rate, application method, fertilizer effects, and phenotypic responses, with few studies examining the relevant mechanisms [8,10,12]. The understanding of N absorption and utilization in blueberries still lacks depth and comprehensiveness.

This study aimed to elucidate the physiological mechanisms underlying nitrogen form regulation in blueberry cultivation. We investigated the effects of three N forms (NH_4_^+^, NO_3_^−^, and urea) on blueberry growth, photosynthesis performance, antioxidant system, carbohydrate metabolism, enzymes involved in N metabolism, free amino acid composition and content, as well as the correlation between these indicators. The findings of this study can provide scientific and realistic fertilization solutions for production practice, with significant implications for enhancing N-use efficiency, reducing production costs, and mitigating environmental pollution.

## 2. Results

### 2.1. Plant Growth Indicators and Carbon (C) and N Content

**Figure 1:**
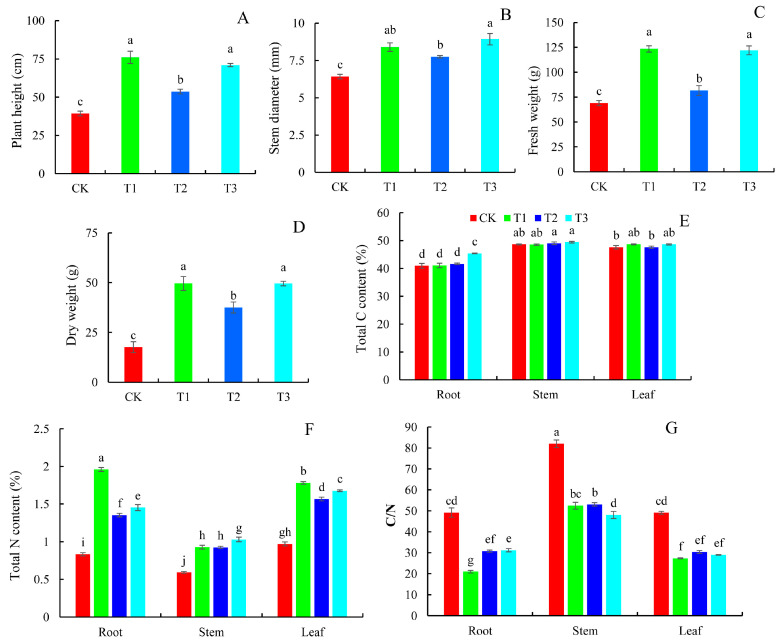
Plant growth indicators and carbon (C) and nitrogen (N) content in blueberries in response to different N forms. (**A**) Plant height; (**B**) stem diameter; (**C**) fresh weight; (**D**) dry weight; (**E**) total C content; (**F**) total N content; and (**G**) C/N ratio. Values are means ± SDs, n = 3. Significant differences (*p* < 0.05) among the four N treatments and among different organs are indicated by different lowercase letters.

The growth of blueberry plants under different N forms was significantly different (Figure S1). The plant height, stem diameter, fresh weight, and dry weight after ammonium-N (T1), nitrate-N (T2), and urea-N (T3) treatments were considerably higher than those with N deficiency (CK) treatment (Figure 1). The promoting effect of T1 and T3 treatments was more obvious, and the increased amount was higher than that of the T2 treatment. In comparison to the T2 treatment, T1 and T3 treatments raised plant height by 41.62% and 32.30%, stem diameter by 8.30% and 15.23%, fresh weight by 51.22% and 49.49%, and dry weight by 32.03% and 31.95%, respectively (Figure 1A–D).

The content of C and N in plants is critical for growth, metabolism, and environmental adaptation. Under different N forms, the total C content was the highest in blueberry stems, followed by leaves and roots (Figure 1E). The total C content of leaves under the T1 treatment was much greater than that of CK and T2, but no statistically significant difference was observed compared to T3. Additionally, the T3 treatment resulted in a moderate increase in root total C content, showing 10.84%, 10.37%, and 9.00% higher values compared to CK, T1, and T2, respectively. It was found that the control group had the lowest total N content in blueberry tissues, followed by the T2 treatment group (Figure 1F). Under the conditions of T1 and T3, the total N content in blueberry tissues was higher. As a result, the total C/N ratio under the CK condition was significantly greater than that in all treatment groups, with stems having the highest C/N ratio, which was consistent with their elevated total C and reduced total N content (Figure 1G).

### 2.2. Photosynthetic Parameters and Chlorophyll Content

**Figure 2:**
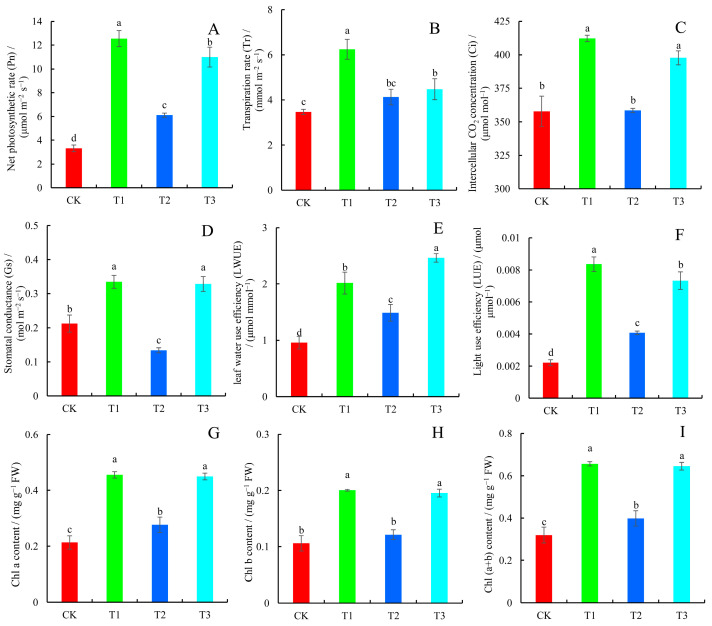
Photosynthetic parameters and chlorophyll content of blueberry leaves in response to different N forms. (**A**) Net photosynthetic rate (Pn); (**B**) transpiration rate (Tr); (**C**) intercellular CO_2_ concentration (Ci); (**D**) stomatal conductance (Gs); (**E**) leaf water-use efficiency (LWUE); (**F**) light-use efficiency (LUE); (**G**) Chl *a* content; (**H**) Chl *b* content; and (**I**) Chl (*a* + *b*) content. Values are means ± SDs, n = 3. Significant differences (*p* < 0.05) among the four N treatments are indicated by different lowercase letters.

Photosynthetic parameters are important indicators of plant growth and stress resistance. T1 treatment was shown to considerably boost blueberry leaf photosynthesis and gas exchange capacity, followed by T3 treatment (Figure 2A–F). The net photosynthetic rate (Pn), transpiration rate (Tr), intercellular CO_2_ concentration (Ci), stomatal conductance (Gs) and leaf light-use efficiency (LUE) of plants under T1 treatment were the highest, and they were 3.8 times, 1.8 times, 1.2 times, 1.6 times, and 3.8 times greater than the control, respectively. T3 treatment resulted in the highest leaf water-use efficiency (LWUE), with an average value of 2.46 µmol mmol^−1^, which increased by 156.86% compared to the control. Similarly, T1 and T3 treatments had the greatest levels of Chl *a*, Chl *b*, and Chl (*a* + *b*), which were significantly greater than in the T2 treatment group and control group (Figure 2G–I). The contents of Chl *a*, Chl *b*, and Chl (*a* + *b*) in the T1 treatment group were greatly elevated by 64.64%, 64.89%, and 64.72%, respectively, compared with the T2 treatment group.

### 2.3. Leaf Ultrastructure

**Figure 3:**
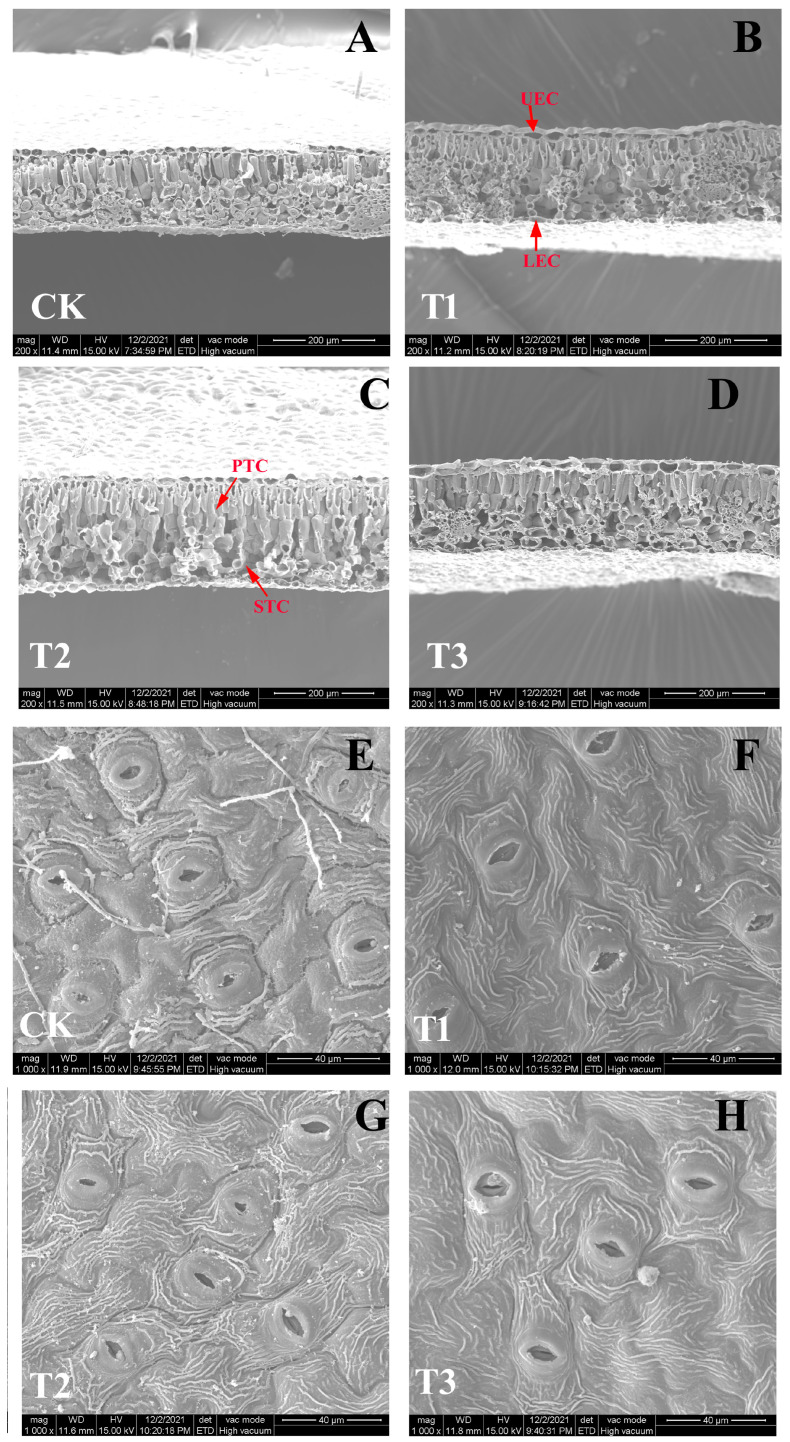
Ultrastructure of blueberry leaves in response to different N forms. Leaf cross-section structure (**A**–**D**); leaf stomatal structure (**E**–**H**). UEC, upper epidermal cell; LEC, lower epidermal cell; PTC, palisade tissue cell; STC, spongy tissue cell.

The findings revealed that differing N forms had considerable effects on the ultrastructure of blueberry leaves. A distinct difference in blade cross-section width was observable in Figure 3A–D, where the control measurement exhibits markedly smaller values than those of the other three treatment groups. The spongy tissue of the T2 treatment group was more loosely organized than that of the other treatment groups. Compared to the leaves of the T1 and T3 treatment groups, the upper and lower epidermal cells in the control and T2 treatment groups were smaller and grew less. The length and width of stomata, as well as stomatal opening, were shown to be substantially smaller in the control and T2 treatment groups than in the T1 and T3 treatments (Figure 3E–H). Furthermore, the control and T2 treatment groups had a more apparent stomatal edge profile than the T1 and T3 treatment groups.

### 2.4. Antioxidant System, Flavonoids, Ellagic Acid, and Sugar Content

**Figure 4:**
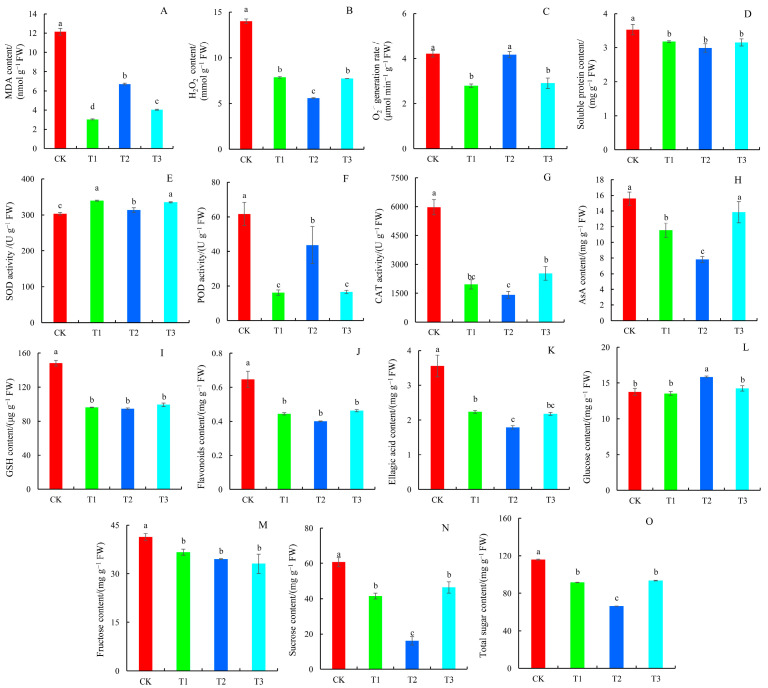
Changes in antioxidant system indexes, flavonoids, ellagic acid, and sugar contents in blueberry leaves in response to different N forms. (**A**) MDA content; (**B**) H_2_O_2_ content; (**C**) the generation rate of O_2_^˙−^; (**D**) soluble protein content; (**E**) SOD activity; (**F**) POD activity; (**G**) CAT activity; (**H**) AsA content; (**I**) GSH content; (**J**) flavonoids content; (**K**) ellagic acid content; (**L**) glucose content; (**M**) fructose content; (**N**) sucrose content; and (**O**) total sugar content. Values are means ± SDs, n = 3. Significant differences (*p* < 0.05) among the four N treatments are indicated by different lowercase letters.

In comparison to the control, MDA, H_2_O_2_, and soluble protein concentrations were reduced considerably. The MDA content of T1 treatment at a minimum plummeted by 75.01% compared to the control (Figure 4A), while C (Figure 4B) and soluble protein content (Figure 4D) were the lowest in T2 treatment, decreasing by 60.06% and 15.29%, respectively. In addition, the production rate of O_2_^˙−^ decreased significantly under T1 and T3 treatments, while there was no significant difference in O_2_^˙−^ production rate during T2 treatment compared to the control (Figure 4C).

As compared to the control, SOD activity significantly increased by 11.94%, 3.45%, and 11.82% from T1 to T3 treatment, respectively (Figure 4E). The activities of POD and CAT enzymes were reduced significantly under different N forms, with POD activity being lowest in the T1 treatment (26.24%) and CAT activity being lowest in the T2 treatment (32.74%) (Figure 4F–G). The AsA content was lowest under the T2 treatment and decreased by 49.87% compared to the control (Figure 4H). GSH and flavonoid levels did not differ substantially between treatments; however, ellagic acid levels were lowest in the T2 treatment, at 80.07% and 82.15% in the T1 and T3 groups, respectively (Figure 4I–K). The glucose content increased slightly, with the highest level found under T2 treatment, with an increase of 15.25% compared to the control (Figure 4L). The contents of fructose, sucrose, and total sugar under different N forms were significantly lower than the control (Figure 4M–O). Although fructose levels did not differ significantly between treatments, the T2 treatment exhibited the lowest sucrose and total sugar concentrations, with reductions of 31.72% and 42.68%, respectively, compared to the control.

### 2.5. Free Amino Acid Content

A total of 17 free amino acids were identified in blueberry leaves in response to different N forms. Among them, histidine (His), isoleucine (Ile), leucine (Leu), lysine (Lys), methionine (Met), phenylalanine (Phe), threonine (Thr), and valine (Val) are the eight essential amino acids (EAA), while aspartic acid (Asp), alanine (Ala), arginine (Arg), cysteine (Cys), glutamic acid (Glu), glycine (Gly), proline (Pro), serine (Ser), and tyrosine (Tyr) are the nine non-essential amino acids (NEAA).

In comparison to the control group, all treatment groups had greater levels of EAA, NEAA, and total amino acid (TAA) (Table 1). The T2 treatment group had the highest levels of EAA, NEAA, and TAA, which were 3.52, 5.60, and 4.82 times higher than in the control group. The amino acid concentration was not significantly different between the T1 and T3 treatment groups. Moreover, under T1 and T3 treatments, Ala was the most abundant amino acid in the leaves, accounting for 15.26% and 13.04% of the total amino acids (Table S1), respectively, followed by Phe, Ser, His, and Pro. Under T2 treatment, the amino acid with the highest concentration in leaves was Arg, which was 120.30 times more than the control, followed by His, Ala, Thr, and Pro. These amino acids account for 49.09%, 8.57%, 7.50%, 5.41%, and 5.39% of the total amino acids, respectively (Table S1).

### 2.6. Key Enzyme Activities Involved in N Metabolism

**Figure 5:**
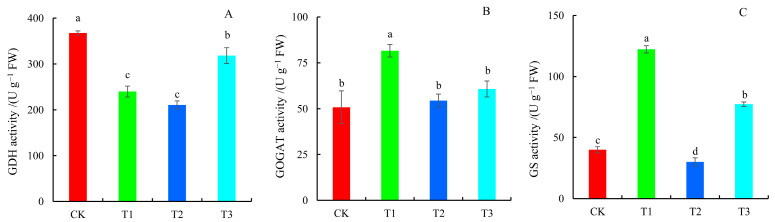
Key enzyme activities involved in the N metabolism of blueberry leaves in response to different N forms. (**A**) GDH activity; (**B**) GOGAT activity; and (**C**) GS activity. Significant differences (*p* < 0.05) among the four N treatments are indicated by different lowercase letters.

The activity of glutamate dehydrogenase (GDH) was significantly lower under all N forms compared to the control, with the lowest activity occurring under T2 treatment, which was reduced by 42.66% compared to the control (Figure 5A). Glutamate synthetase (GOGAT) activity increased across all treatment groups compared to the control. GOGAT activity increased by 60.76% in the T1 treatment group; nevertheless, there was no significant difference between the T2 and T3 treatment groups (Figure 5B). In addition, the glutamine synthetase (GS) activity of the T1 and T3 treatment groups was 3.05 and 1.93 times higher than that of the control group, respectively, whereas the GS activity of the T2 treatment group was substantially lower than that of the control group, decreasing by 24.91% (Figure 5C).

### 2.7. Physicochemical Properties of Blueberry Cultivation Substrate

The physicochemical parameters of blueberry growth substrates varied greatly depending on the N forms used (Table 2). Compared to the control, T1 treatment greatly reduced the pH value of the cultivated substrate, followed by the T3 treatment group. However, T2 treatment significantly increased the pH value of the substrate. The EC value of the culture substrate in the T1 treatment was 3.47 times that of the control group. The T3 treatment had the highest organic matter and organic carbon content compared to the control group, followed by T1 treatment. Furthermore, T1 treatment resulted in the greatest concentration of AHN in the culture substrate, which was 4.30 times higher than the control.

### 2.8. Correlation and Principal Component Analysis (PCA) of Physiological Parameters

**Figure 6:**
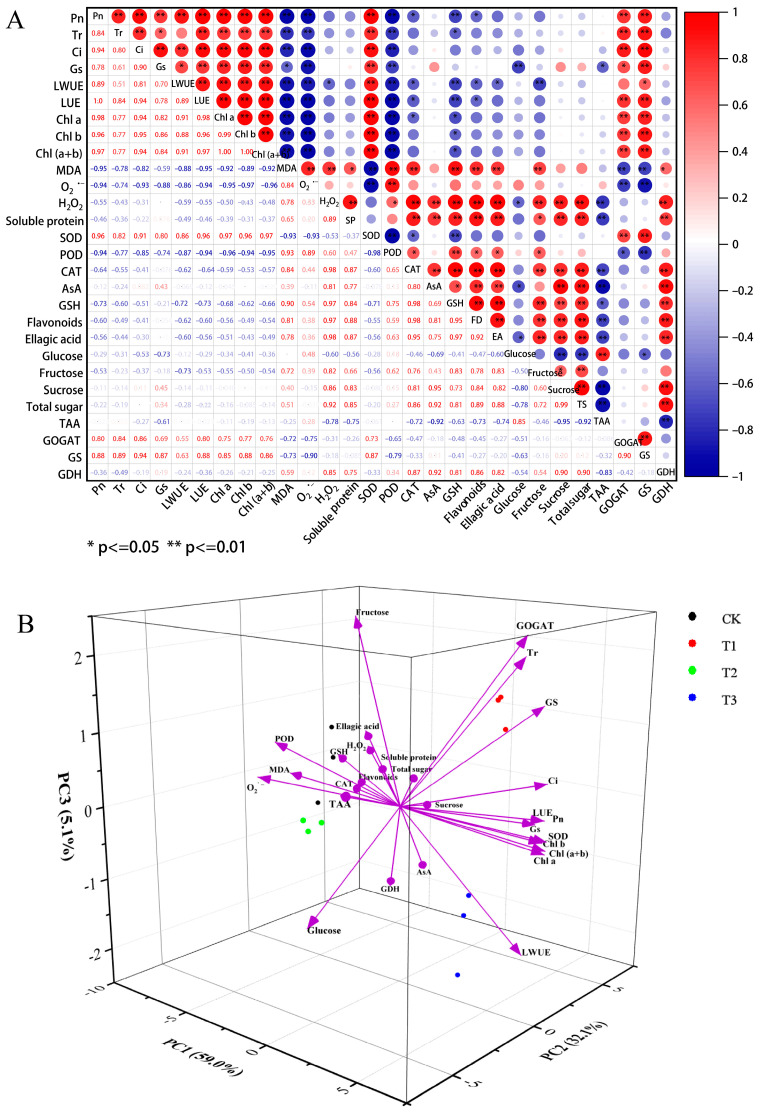
(**A**) Correlation matrix of different physiological indexes in blueberry plants. A significant correlation is denoted by the symbols * and ** at the 0.05 and 0.01 levels, respectively. SP, Soluble protein; FD, Flavonoids; EA, Ellagic acid; TS, Total sugar. (**B**) PCA score plots of different physiological indexes in blueberry plants in response to different N forms.

The correlation analysis results revealed that there was no significant relationship between Tr and LWUE, or between SOD and LWUE. Pn, Tr, Ci, Gs, LWUE, LUE, Chl *a*, Chl *b*, and Chl (*a* + *b*) content, as well as GS, GOGAT, and SOD activity, all showed substantially or highly significant positive correlations (Figure 6A). MDA content, the generation rate of O_2_^˙−^, and POD activity had a substantial negative correlation with photosynthetic parameters, chlorophyll content, and GS and GOGAT activities. H_2_O_2_ and soluble protein levels have a strong positive correlation with CAT activity, AsA, GSH, flavonoids, ellagic acid, fructose, sucrose, and total sugar contents. TAA levels were positively correlated with glucose content but negatively correlated with the majority of other antioxidant indicators. Moreover, correlation analysis between each amino acid and other physiological indicators showed that Asp, Ile, Leu, Tyr, and Val exhibited significant positive correlations with the growth indicators Pn, Tr, Ci, LWUE, LUE, Chl *a*, Chl *b*, and Chl (*a* + *b*) content (Figure S2). Interestingly, GDH activity is associated positively with the majority of antioxidant indicators.

The PCA results revealed that the eigenvalues of the two principal components, PC1 and PC2, were both greater than 1, explaining 59.03% and 32.10% of the variation, respectively, with a total cumulative variance contribution of 91.13% (Figure 6B, Table 3). It is clear that these two main components can accurately cover the information of various physiological indicators, and data reliability is excellent. Furthermore, the treatments had substantial differences, and the scatter points were clearly separated (Figure 6B). The loading findings of PC1 revealed that photosynthetic parameters, chlorophyll content, and GS, GOGAT, and SOD activities had significant positive contributions to PC1, but antioxidant system indexes (MDA, O_2_^˙−^, H_2_O_2_, POD, CAT, GSH, flavonoids, and ellagic acid) and soluble protein and fructose content had significant negative effects. The PC2 loading results revealed that GS, GDH activity, AsA, sucrose, and total sugar content had a significant positive impact on PC2, while glucose and TAA content had a substantial adverse contribution (Table 3).

## 3. Discussion

### 3.1. Plant Nitrogen Form Preference

Nitrogen (N) is one of the indispensable elements for plant growth and development [3], which plays a pivotal role in plant metabolism, photosynthesis, dry matter transport and distribution, as well as the formation of yield and quality. Low N utilization efficiency can cause significant alterations in plant physiological and biochemical processes. Improving plant N absorption and assimilation capacity is critical in sustainable agricultural production for high crop output and reduced environmental risks [13]. Previous research has found that the inorganic N sources that higher plants can absorb and utilize are primarily nitrate-N and ammonium-N, whereas organic N sources are primarily amide-N and amino acids. Among them, nitrate-N is the main source of N absorbed and utilized by the vast majority of plants compared to ammonium-N [14]. Plants’ long-term evolution has resulted in a selective preference for various types of N, which significantly impacts their productivity and N usage efficiency [5,12]. Numerous studies have revealed that *Arabidopsis* prefers to absorb nitrate-N and is more susceptible to ammonium-N [15]. Rice (*Oryza sativa* L.) prefers ammonium-N, and wheat (*Triticum aestivum* L.) prefers nitrate-N [16]. Cabbage (*Brassica pekinensis* L.) prefers organic N over amide-N [17]. Some scholars believe that supplying ammonium-N to plants such as blackberries (*Rubus* spp.), tea (*Camellia sinensis* L.), and coffee (*Coffea arabica* L.) can enhance their N metabolism [18,19,20]. Although the mechanism of N metabolism in *Arabidopsis*, rice, wheat, and other woody plants has been extensively explored, the mechanism of N absorption preference in blueberries is still poorly understood [12]. Most studies have shown that blueberries prefer to absorb ammonium-N [8], whereas others have shown little preference for N. Among them, Rosen et al. found that, regardless of the N source supplied, blueberries grow well and have high biomass under soil conditions with a pH of 4.5. This may be related to the variety of blueberries [21]. Alt et al. showed that blueberries could assimilate nitrate-N very efficiently when it was supplied directly to the cut ends of the stem [22]. In view of this, this experiment studied the internal mechanism of the influence of different forms of N on the growth and development of blueberries under isonitrogen conditions, with the goal of providing technical support and a theoretical foundation for the precise fertilization and efficient N fertilizer utilization in the blueberry seedling stage.

### 3.2. Growth Status of Blueberries in Response to Different N Forms

The external morphology of plants can directly reflect the effects of different N forms on plant growth status. This study found that the plant height and stem diameter in the T1 and T3 treatment groups were considerably greater than those in the control group under three N treatments: ammonium-N (T1), nitrate-N (T2), and amide-N (T3). The plant height and stem diameter in the T2 treatment group were considerably greater than in the control group but lower than in the T1 and T3 treatment groups. Biomass is the consequence of plant dry matter accumulation which, in some cases, shows plant growth state and development potential [5]. Guo et al. found that different inorganic N forms significantly influence the biomass allocation of buffalograss [23]. Under nitrate-N treatment, buffalograss seedlings exhibited higher biomass, indicating a preference for the uptake and utilization of nitrate-N. In this study, our results discovered that the fresh and dry weights of blueberry seedlings in the T1 and T3 treatment groups were notably higher than those in the T2 treatment and the control groups. These results indicate that ammonium-N and amide-N treatments significantly enhanced the growth performance of blueberry plants. The C and N content of plants, along with the C/N ratio, have a direct impact on N uptake and utilization efficiency [3]. Generally, a higher C/N ratio favors the N resources provided by protein decomposition for young leaf growth, hence driving leaf senescence [24]. In this study, the control group had lower N content and a higher C/N ratio than the other N forms of treatments. This indicates that N deficiency limits the synthesis of N-containing compounds such as proteins and nucleic acids, prevents the effective conversion of carbon assimilation products into nitrogenous compounds, and accelerates blueberry leaf senescence. Araya et al. also demonstrated that the leaves of *Phaseolus vulgaris* L. acquired considerably more carbohydrates at low N nutrient levels [25]. Our results also showed that the fructose, sucrose, and total sugar content in the control leaves were significantly higher compared to the other treatment groups. In addition, the C/N ratio of leaves in the T2 treatment group was noticeably greater than that in the T1 and T3 treatment groups, suggesting that nitrate-N treatment was not conducive to the growth of blueberries to a certain extent. It is evident that in agricultural production, the rational selection of N fertilizer forms may contribute to optimizing blueberry growth and yield.

### 3.3. Photosynthetic Performance and Antioxidant Capacity of Blueberries in Response to Different N Forms

Plant growth and development rely on photosynthesis for material and energy [26]. The molecules involved in photosynthesis, such as proteins, different coenzymes, and chlorophyll, all contain N, hence plant photosynthetic efficiency is bound to be influenced by plant N-use efficiency [26]. Moreover, numerous studies have demonstrated that different N forms significantly affect plant photosynthesis, with nitrate-N treatment typically resulting in higher photosynthetic rates [27,28]. In this study, it was found that the net photosynthetic rate (Pn), transpiration rate (Tr), intercellular CO_2_ concentration (Ci), stomatal conductivities (Gs), and leaf light-use efficiency (LUE) in the T1 treatment group were the highest, indicating that plants had stronger photosynthesis and higher N-use efficiency under this treatment condition. This finding is also consistent with the good stomatal condition found in the ultrastructure of blueberry leaves. Cao et al. revealed that when treated with ammonium-N, *Lonicera japonica* exhibited diminished photosynthetic ability mainly due to three anatomical changes: shrinkage of intercellular spaces, reduction in chloroplast quantity, and thickening of cell walls [27]. These findings highlight the significant role of leaf anatomical modifications in mediating photosynthetic responses to varying N sources. Chlorophyll content, being a critical pigment for plant photosynthesis, can partially indicate the level of photosynthetic rate [29]. Plants in the T1 and T3 treatment groups had significantly higher chlorophyll contents than both the T2 treatment group and the control group. This suggested that the plants in the two groups had a higher photosynthetic rate, which was also consistent with the actual photosynthetic rate results.

Reactive oxygen species (ROS) are key regulatory factors involved in plant metabolism and stress resistance. Excess ROS can be hazardous to the plant and trigger its defense system. Plants maintain ROS homeostasis primarily through enzymatic and non-enzymatic antioxidant systems [30]. Different N forms influence the plant antioxidant system by dynamically regulating the balance between ROS generation and their detoxification [31,32]. In this study, SOD and CAT activities, AsA, ellagic acid, sucrose, and total sugar contents in plant leaves under T1 and T3 treatment were significantly higher than those in the T2 treatment group, whereas MDA content and O_2_^˙−^ production rates were substantially lower. The results showed that ammonium-N and amide-N treatments could help plants maintain their normal physiological function by increasing the levels of antioxidant enzymes, antioxidant substances, and osmotic regulatory substances like soluble protein and soluble sugar in leaves, as well as improving plant stress resistance. Hessini et al. also found that ammonium-N-fed *Spartina alterniflora* plants had higher antioxidant enzyme activity as compared with nitrate-N-fed plants [31]. Similarly, Duan et al. investigated the impact of different N forms on blackberry fruit quality, revealing that ammonium-N treatment significantly enhanced total antioxidant activity, DPPH radical scavenging capacity, and the content of ellagic acid, flavonoids, and VC [32]. POD predominantly eliminates H_2_O_2_ generated in cells by converting it to water [33]. The increased POD activity in the T2 treatment group could be the primary cause of the lower H_2_O_2_ levels. Additionally, the control group had higher levels of ROS, antioxidant enzyme activity, antioxidant substances, soluble protein, fructose, sucrose, and total sugar, indicating that the structure and function of plant cell membranes were severely damaged under N deficiency conditions, and the generated antioxidant enzymes and antioxidant substances were unable to clear excess ROS in time. In barley, N deficiency increased leaf antioxidant enzymes, such as ascorbate peroxidase (APX), peroxidase (POD), and catalase (CAT), and root antioxidant enzymes (APX and POD) [34]. N deprivation in *Oryza sativa* leaves also raised the activity of antioxidant enzymes and the levels of antioxidant molecules [35].

### 3.4. Free Amino Acids Changes in Blueberries in Response to Different N Forms

As a significant form and primary transport form of N assimilates in plants, the level of free amino acids can represent the state as well as the effectiveness of N supply in plants to some extent [36]. The study found that the free amino acid content of plant leaves under various N forms was significantly higher than that in the control group, with the T2 treatment group having the highest amount, followed by the T1 and T3 treatments. Among these, the T2 treatment group had the greatest Arg level, reaching 79.40 mg 100 g^−1^, which was 26 and 24 times that of the T1 and T3 treatment groups, respectively. This was also the main reason for the highest free amino acid content in this treatment group. Previous research has shown that Arg, as a N storage carrier, is also a precursor of polyamines and nitric oxide, which are catalyzed by key enzymes and participate in signal regulation of many important biochemical reactions in plants, including growth and development and stress protection [37]. The greater Arg level in the T2 treatment group could play an essential role in ROS elimination in plant leaves. In addition to Arg, the leaves of all treatment groups had rather high levels of Ala, Pro, and His. Ala is strongly related to pyruvate metabolism [38] and accumulates significantly under various N forms, implying that it may serve as a metabolic foundation for plants to adjust to environmental changes by boosting gluconeogenesis. As a highly effective antioxidant, a rise in free Pro concentration could serve a crucial role in regulating the equilibrium of ROS in plant cells across all treatment groups, safeguarding biofilm integrity, and maintaining protein-advanced structure [39]. Moreover, in the T1 and T3 treatment groups, Phe acts as a polyphenol precursor [40], and enhancing its metabolism may reduce ROS oxidative damage and maintain cell homeostasis in plants by stimulating secondary metabolic pathways. According to Mao et al., ammonium-N treatment not only significantly increased the total amino acid content in mini Chinese cabbage but also resulted in a significantly higher Phe content compared to nitrate-N treatment [17]. It has been reported that applying His externally might considerably improve maize photosynthetic efficiency, enzyme activity, and gene expression during salt stress [41]. In this study, greater His content may serve a regulatory role in increasing plant photosynthetic rate. Furthermore, due to the limitations of current analytical techniques, the detection of glutamine (Gln) and asparagine (Asn) in the leaves may have been compromised in this study. Consequently, their potential functional roles in blueberries’ adaptation to different N forms could be underestimated. In the subsequent research, we will refine amino acid extraction protocols and enhance detection sensitivity, enabling precise tracking of their dynamic changes. Additionally, functional genomic investigations targeting N metabolism-related genes will be conducted to elucidate the mechanistic contributions of these amino acids.

### 3.5. Enzymes Involved in N Metabolism of Blueberries in Response to Different N Forms

In higher plants, N absorption is primarily performed via the GS/GOGAT cycle [42,43]. Ma et al. showed that increasing GS and GOGAT activities can reduce ammonium toxicity and boost N assimilation, hence enhancing plant development [42]. This study showed that the activities of GS and GOGAT in the T1 treatment group were greatly improved compared to the control and T2 treatment groups, indicating that ammonium-N treatment promoted N metabolism in plants, whereas nitrate-N treatment inhibited N metabolism. Under normal growth conditions, GDH in most species primarily catalyzes the deamination of glutamate to form α-ketoglutarate, which acts as an amino acceptor in transamination during amino acid degradation [44]. Notably, *CsGDHs* and *CsGSs* in tea plants have been shown to synergistically assimilate ammonium into glutamine [45]. Our findings suggested that elevated GDH activity under N deficiency may participate in N remobilization in leaves, balancing N utilization and energy supply, thereby improving plant environmental adaptability [44,45,46]. Additionally, whether GDH in blueberries, which share similar N preferences with tea plants [19], collaborates in ammonium assimilation requires further experimental validation.

### 3.6. Growth Substrate Effect

Additionally, the quality of the soil environment directly influences whether plants can absorb enough nutrients, affecting plant growth and development [47]. In this study, blueberry plants grew better under T1 and T3 treatments. The physicochemical parameters of the growth medium were investigated, and it was discovered that the two treatments had lower pH values, higher EC values, and AHN content. It is clear that these two treatments offer the plants more mineral nutrients, particularly N, and that the low pH value creates a more acidic environment conducive to blueberry growth. The findings also demonstrated that N forms significantly influence substrate organic matter and organic carbon, with T3 showing the highest levels, followed by T1, highlighting their importance for blueberry growth [48]. The amide-N treatment (T3) likely enhances organic matter and organic carbon content by promoting microbial activity and plant growth, thereby increasing plant and microbial residue accumulation. Previous research suggests that amide-N supplies energy and substrates for microorganisms, accelerating organic matter decomposition [49], while also enhancing microbial activity via root exudates, fostering organic carbon accumulation [50].

## 4. Materials and Methods

### 4.1. Plant Material

Two-year cuttings of the blueberry cultivar ‘Anna’ were used as the experimental material and cultivated in the greenhouse of Institute of Botany, Jiangsu Province and Chinese Academy of Sciences (32°10.01″ N, 118°49′58.22″ E). In the experiment, pure coconut coir (C/N 80) was selected as the growing medium (Shanghai Kangkang International Trade Co., Ltd., Shanghai, China). Healthy plants with similar shapes and sizes were transplanted into plastic pots (upper diameter 30 cm, lower diameter 24 cm, height 33 cm), one plant per pot. After 40 days of adaptive pre-culture and 20 days of fertilizer control, seedlings were fed with various N forms, including N deficiency (CK), ammonium-N (T1), nitrate-N (T2), and amide-N (T3). The experiment employed a completely randomized design, with three biological replicates per treatment and three plants per replication. Each treatment nutrient solution contained 5 mM KCl, 1 mM KH_2_PO_4_, 2 mM MgSO_4_·7H_2_O, 0.09 mM EDTA·FeNa, 0.07 mM KI, 0.05 mM H_3_BO_3_, 0.1 mM MnSO_4_·H_2_O, 0.03 mM ZnSO_4_·7H_2_O, 1 µM Na_2_MoO_4_·2H_2_O, and 1 µM CuSO_4_·5H_2_O. In addition, the control group contained 4 mM CaCl_2_·2H_2_O but did not contain N. The total nitrogen supply concentration in the three treatments was the same. The ammonium-N group contained 7.5 mM (NH_4_)_2_SO_4_ and 4 mM CaCl_2_·2H_2_O. The nitrate-N group contained 4 mM Ca(NO_3_)_2_·4H_2_O and 7 mM NaNO_3_. The amide group contained 4 mM CaCl_2_·2H_2_O and 7.5 mM CO(NH_2_)_2_. During the experiment, the nutrient solution was poured weekly, with a quantity of 300 mL per basin each time, and was treated continuously for three months, during which water was replenished in time according to the water loss situation.

### 4.2. Measurement of Plant Material Growth Indexes, Total C and Total N Content

A tape rule and a digital vernier caliper were used to measure the plant height and stem diameter, respectively. The whole plant samples were taken for biomass analysis. The plants were cleaned with distilled water and dried with filter paper before being weighed on a balance with an accuracy of 0.01 g to determine their fresh weight. The plant samples were initially dried at 80 °C for 30 min before being dried at 105 °C to a constant weight in the oven. Dry weight was determined from the dried samples. The total C and total N contents were measured using a PE2400II element analyzer (PE, Cincinnati, OH, USA).

### 4.3. Measurement of Photosynthetic Parameters and Chlorophyll Content

On July 17th, the photosynthetic parameters were obtained by a photosynthesizer (LI-6800, Beijing Ligotai Technology Co., Ltd., Beijing, China). Measurements were taken between 8:00 and 11:00 a.m. at a light intensity of around 1500 µmol (m^2^ s)^−1^. Five mature leaves from healthy branches of each plant were chosen for analysis. The amount of chlorophyll was evaluated using the ethanol extraction method [51].

### 4.4. Analysis of Antioxidant System Indexes

The malondialdehyde (MDA) content was measured using the thiobarbiturate (TBA) method [52]. The formation rate of superoxide anion radical (O_2_^˙−^) was evaluated using the hydroxylamine oxidation reaction method [53]. The content of hydrogen peroxide (H_2_O_2_) was determined using the kit from Nanjing Jiancheng Biological Co., Ltd., Nanjing, China. The content of soluble protein was determined by the coomassie bright blue staining method [54]. The activity of superoxide dismutase (SOD) was assessed using the nitronitrogen blue tetrazole (NBT) method [55]. Peroxidase (POD) activity was determined based on guaiacol oxidation [56]. Catalase (CAT) activity was quantified using the ammonium molybdate colorimetric assay [57]. The content of ascorbic acid (AsA) was determined by Law et al. [58]. The content of reduced glutathione (GSH) was determined by the 5,5′-Dithio-BIS-(2-nitrobenzoic acid) (DTNB) method [51].

### 4.5. Determination of Sucrose, Fructose and Glucose Content

The contents of sucrose, fructose, and glucose were determined with the kits of Nanjing Jiancheng Biological Co., Ltd. The total sugar amount was calculated as the sum of the three sugar contents.

### 4.6. Determination of Flavonoids and Ellagic Acid Content

The total flavonoids content was calculated by the national standard GBT205742006. First, 3 g of fresh samples were homogenized in 30 mL 95% ethanol, then heated in a water bath at 65 °C for 45 min, centrifuged at 25 °C 6000 r min^−1^ for 10 min, and the supernatant was obtained. An amount of 3 mL of supernatant was transferred into a clean tube, and 1 mL of 100 g L^−1^ Al(NO_3_)_3_, 10 mL of 95% ethanol, and 1 mL of 9.8 g L^−1^ CH_3_COOK were added. After keeping at room temperature for 1 h, the absorbance was measured at 415 nm.

The ellagic acid content was determined by the method described by Maas et al. [59]. Fresh samples (3 g) were homogenized in 30 mL of 40% ethanol. The homogenates were extracted ultrasonically at 80 °C for 20 min and centrifuged at 25 °C for 6000 r min^−1^ for 10 min to obtain the supernatant. Then, 1 mL of supernatant was mixed with 4 mL of 0.1 mol L^−1^ NaOH solution, and the solution developed a blue color after a full reaction for 15 min. The absorbance was measured at 357 nm.

### 4.7. Determination of Free Amino Acid Content

Sample preparation: A 0.2 g dry leaf sample was ground into powder, filtered to 0.25 mm, and placed in a conical bottle containing 10 mL of boiling water. It was simultaneously heated and oscillated for 20 min in a water bath set at 95 °C. Following filtering and vacuum freeze-drying (FD5 freeze-drying equipment, SIM, Charlotte, NC, USA), the extract was diluted in dilution buffer and filtered through a 0.2 µm membrane. Sample determination: The sample was evaluated using an S443D automatic amino acid analyzer (Secam Scientific Instruments Co., Ltd., Eresing, Germany). The chromatography was performed on the LCAK06/Na column. The mobile phases A and B consisted of 0.012% citric acid–sodium citrate (pH 3.45) and 0.02% citric acid–sodium citrate (pH 10.85), respectively. The measurement was carried out using a gradient temperature control of 58–74 °C. The flow rates for the eluting pump and the derivative pump were set at 0.45 mL min^−1^ and 0.25 mL min^−1^, respectively. The ultraviolet detection wavelengths were 570 and 440 nm [60].

### 4.8. Determination of Glutamate Dehydrogenase (GDH), Glutamine Synthetase (GS), and Glutamate Synthetase (GOGAT) Activities

The GDH activity was determined by the GDH kit (BC1460, Beijing Solarbio Science and Technology Co., Ltd., Beijing, China). GDH catalyzes the conversion of NH_4_^+^, α-ketoglutarate, and NADH into glutamate and NAD^+^, leading to a decrease in absorbance at 340 nm. GDH activity was determined by measuring the absorbance at 340 nm.

The GS and GOGAT activities were measured using the GS and GOGAT kits (BC0910 and BC0070, Beijing Solarbio Science and Technology Co., Ltd.). GS catalyzes the formation of glutamine from ammonium ions and glutamate, utilizing ATP and Mg^2+^ as cofactors. Glutamine is transformed to γ-glutamyl hydroxamic acid, which forms a red complex with iron under acidic conditions. GS activity was assessed by measuring the absorbance at 540 nm. GOGAT employs NADH as an electron donor to catalyze the transfer of glutamine’s amino group to α-ketoglutarate. The changes in the absorbance at 340 nm can indicate the magnitude of GOGAT activity.

### 4.9. Micromorphology Observation of Blueberry Leaf

The leaf ultrastructure was observed according to the method of Dong et al. [61]. First, the sample was washed with 0.2 mM potassium phosphate buffer (pH 7.2), then cut into tiny pieces (1.5 × 2 mm) and fixed in 4% (*v*/*v*) glutaraldehyde. After further dehydration and freeze-drying in different ethanol and tert-butanol concentrations, the samples were gilded using an ion-sputtering device. The observation was conducted with a Quanta 200 environmental scanning electron microscope (FEI, Enschede, The Netherlands).

### 4.10. Measurement of the PH and Conductivity (EC) Values of the Substrate

The pH and EC values of the substrate were measured with a pH meter and a conductivity meter, respectively. Preparing the supernatant: After dissolving 1 g of dry substrate in 10 mL of water, centrifuge at 8000 rpm at 4 °C for 10 min. The supernatant can be utilized for detection.

### 4.11. Measurement of Organic Matter and Alkali-Hydrolysable Nitrogen Content

The organic matter content of the substrate was measured using the potassium dichromate volumetric method. The 0.01 g dry substrate was combined with 5 mL of concentrated sulfuric acid and 5 mL of 0.8 mol L^−1^ 1/6 K_2_CrO_7_ standard solution in 50 mL glass test tube. Then the mixture was heated to 180 °C and titrated with 0.2 mol L^−1^ FeSO_4_ after 5 min. The details can be found in an earlier study [18].

The alkaliolytic diffusion method was employed to quantify alkali-hydrolysable nitrogen (AHN) in the substrate. The 0.5 g dry substrate was carefully weighed and equally placed in the diffusion dish, followed by 10 mL of sodium hydroxide hydrolyzed substrate (1.2 mol L^−1^). After diffusion, NH_3_ was absorbed with 2 mL of 2% boric acid solution and titrated with 0.01 mol L^−1^ standard hydrochloric acid solution. The details can be acquired from earlier research [18].

### 4.12. Statistic Analysis

The experimental data were statistically analyzed using SPSS 20.0 and Microsoft Office Excel 2016. Treatment differences were analyzed using one-way ANOVA (Tables S2–S7) and Tukey’s test, with significance determined at *p* < 0.05. In the analysis, it was found that, with the exception of Glu (Table S4), the *p*-values for all other indicators were less than 0.05. This demonstrates that the intergroup differences were statistically significant, and the results hold practical research significance. Origin 2022 (Origin Lab Inc., Northampton, MA, USA) software was used to perform correlation and principal component analyses of the data.

## 5. Conclusions

In conclusion, N forms differentially regulate blueberry growth and development. Juvenile blueberry plants exhibit preferential uptake of ammonium-N (NH_4_^+^) and amide-N (urea), which significantly enhance plant height and biomass accumulation, chlorophyll content and photosynthetic efficiency, total nitrogen and amino acid contents, activities of key enzymes (SOD, GS, GOGAT), and antioxidant capacity (AsA, flavonoid, ellagic acid, sucrose, and total sugar). In contrast, nitrate-N (NO_3_^−^) treatment led to the accumulation of reactive oxygen species (O_2_^˙−^, H_2_O_2_) and increased malondialdehyde (MDA) content. The elevated POD activity and Arg content under nitrate-N treatment may represent an adaptive stress response. These findings suggest an optimized fertilization strategy for blueberry cultivation during vegetative growth: higher ammonium-N but reduced nitrate-N application.

## Figures and Tables

**Table 1:** Free amino acid contents in blueberry leaves in response to different N forms.

Amino Acid (mg(100 g)^−1^ DW)	CK	T1	T2	T3
Asp	1.74 ± 0.06 c	5.03 ± 0.06 a	3.61 ± 0.42 b	3.35 ± 0.82 b
Ala	7.16 ± 0.24 b	11.43 ± 0.34 a	12.14 ± 0.96 a	7.67 ± 1.91 b
Arg	0.66 ± 0.25 b	3.02 ± 0.08 b	79.40 ± 5.99 a	3.30 ± 0.88 b
Cys	0.22 ± 0.04 c	0.46 ± 0.18 bc	1.00 ± 0.08 a	0.80 ± 0.18 ab
Glu	2.73 ± 0.16 a	1.95 ± 0.90 a	2.03 ± 0.32 a	1.64 ± 0.58 a
Gly	0.34 ± 0.02 b	0.62 ± 0.07 b	0.98 ± 0.06 a	0.61 ± 0.16 b
His	2.32 ± 0.12 d	7.48 ± 0.16 b	13.86 ± 1.09 a	5.04 ± 0.50 c
Ile	1.81 ± 0.29 b	3.02 ± 0.11 a	2.18 ± 0.16 ab	3.17 ± 0.78 a
Leu	1.70 ± 0.01 b	3.39 ± 0.18 a	2.19 ± 0.17 ab	3.62 ± 0.89 a
Lys	1.34 ± 0.05 c	3.10 ± 0.12 b	6.19 ± 0.50 a	2.69 ± 0.65 b
Met	0.03 ± 0.0046 bc	0.01 ± 0.0003 c	0.06 ± 0.0061 a	0.04 ± 0.0163 ab
Phe	2.57 ± 0.14 b	8.46 ± 0.64 a	7.96 ± 0.34 a	6.69 ± 1.70 a
Pro	3.66 ± 0.36 b	6.58 ± 0.80 ab	8.71 ± 0.71 a	6.57 ± 2.16 ab
Ser	2.73 ± 0.42 c	8.22 ± 0.56 a	6.29 ± 1.24 ab	5.38 ± 1.64 bc
Tyr	1.92 ± 0.08 c	5.29 ± 0.08 a	2.64 ± 0.30 bc	3.41 ± 0.84 b
Thr	1.18 ± 0.13 b	2.23 ± 0.10 b	8.75 ± 1.03 a	2.00 ± 0.52 b
Val	2.39 ± 0.09 b	4.62 ± 0.06 a	3.76 ± 0.27 ab	4.55 ± 1.09 a
∑EAA	12.76 ± 1.00 c	32.30 ± 1.25 b	44.94 ± 3.48 a	26.10 ± 7.31 b
∑NEAA	20.83 ± 0.86 c	42.60 ± 1.92 b	116.81 ± 8.07 a	32.73 ± 6.82 bc
∑TAA	33.59 ± 1.84 c	74.90 ± 3.11 b	161.75 ± 11.47 a	58.83 ± 14.12 b

Values are means ± SDs, n = 3. Significant differences (*p* < 0.05) among the four N treatments are indicated by different lowercase letters. Total amount of the eight essential amino acids (∑EAA); Total amount of the nine non-essential amino acids (∑NEAA). Total amino acids content (∑TAA): ∑EAA + ∑NEAA.

**Table 2:** Physicochemical parameters of blueberry cultivation substrates in response to different forms of N treatments.

Treatment	pH	EC (mS cm^−1^)	Organic Matter (%)	Organic Carbon (%)	Alkali-Hydrolyzable N (AHN) (mg kg^−1^ DW)
CK	5.49 ± 0.06 b	0.97 ± 0.04 d	73.02 ± 0.75 c	42.35 ± 0.44 c	368.67 ± 15.42 c
T1	4.42 ± 0.0 d	3.37 ± 0.02 a	74.94 ± 0.70 b	43.47 ± 0.40 b	1585.73 ± 30.84 a
T2	5.97 ± 0.01 a	2.12 ± 0.02 c	73.56 ± 1.15 bc	42.67 ± 0.67 bc	483.47 ± 4.28 b
T3	4.57 ± 0.02c	2.29 ± 0.01 b	79.84 ± 0.52 a	46.31 ± 0.30 a	516.13 ± 11.66 b

Values are means ± SDs, n = 3. Significant differences (*p* < 0.05) among the four N treatments are indicated by different lowercase letters.

**Table 3:** Eigenvalues and loading coefficients of each principal component.

Parameter	Component	
	1	2
Pn	0.940 **	0.317
Tr	0.794 **	0.257
Ci	0.818 **	0.536
Gs	0.578	0.787 **
LWUE	0.869 **	0.210
LUE	0.940 **	0.317
Chl *a*	0.914 **	0.376
Chl *b*	0.881 **	0.449
Chl (*a* + *b*)	0.906 **	0.397
MDA	−0.999 **	−0.020
O_2_^˙−^	−0.818 **	−0.535
H_2_O_2_	−0.788 **	0.607
Soluble protein	−0.668 **	0.611
SOD	0.918 **	0.336
POD	−0.924 **	−0.250
CAT	−0.850 **	0.506
AsA	−0.417	0.829 **
GSH	−0.909 **	0.404
Flavonoids	−0.823 **	0.534
Ellagic acid	−0.785 **	0.575
Glucose	0.000	−0.944 **
Fructose	−0.714 **	0.377
Sucrose	−0.424	0.893 **
Total sugar	−0.525	0.839 **
TAA	0.277	−0.935 **
GOGAT	0.734 **	0.360
GS	0.730 **	0.602
GDH	−0.616	0.665 **
Total	16.53	8.99
% of variance	59.03	32.10
Cumulative %	59.02	91.13

** Represents eigenvalues that are significant ≥ 0.60.

## Data Availability

The data generated for this study are available on request to the corresponding author.

## Supplementary Materials

The following supporting information can be downloaded at https://www.mdpi.com/article/10.3390/plants14101444/s1, Figure S1: Image showing the external appearance of blueberry plants under different N form treatments; Figure S2: Correlation analysis between each amino acid and other physiological indicators in blueberry plants. A significant correlation is denoted by the symbols *, **, and *** at the 0.05, 0.01, and 0.001 levels, respectively. Table S1: Percentage of individual amino acids relative to total amino acids in response to different N forms; Table S2: ANOVA analysis of blueberries’ growth indicators and carbon (C) and nitrogen (N) content; Table S3: ANOVA analysis of blueberries’ photosynthetic parameters and chlorophyll content; Table S4: ANOVA analysis of blueberries’ antioxidant system indexes, flavonoids, ellagic acid and sugar contents; Table S5: ANOVA analysis of blueberries’ free amino acid content; Table S6: ANOVA analysis of blueberries’ key enzyme activities involved in the N metabolism; Table S7: ANOVA analysis of blueberry cultivation substrates’ physicochemical parameters.
