# Supplementary material for: Growth and Physiological Traits of Blueberry Seedlings in Response to Different Nitrogen Forms
Source: Plants (Basel). 2025 May 12;14(10):1444. doi: 10.3390/plants14101444 (PMC12114734; doi:10.3390/plants14101444)
Supplement: Supplementary file 1 [file plants-14-01444-s001.zip › Supplementary Table S1 (Percentage of individual amino acids).pdf]

Supplementary Table S1 Percentage of individual amino acids relative to total amino acids in response to different N forms.

% of the total amino acids content	CK	T1	T2	T3
Asp	5.19±0.16a	6.71±0.09a	2.23±0.26b	5.70±1.39a
Ala	21.31±0.72a	15.26±0.46b	7.50±0.59c	13.04±3.25b
Arg	1.96±0.74b	4.03±0.11b	49.09±3.70a	5.61±1.50b
Cys	0.66±0.12b	0.62±0.24b	0.62±0.05b	1.36±0.30a
Glu	8.13±0.48a	2.60±1.21b	1.26±0.20b	2.78±0.98b
Gly	1.00±0.07a	0.82±0.09ab	0.61±0.04b	1.04±0.27a
His	6.90±0.34b	9.99±0.22a	8.57±0.67a	8.57±0.85a
Ile	5.38±0.85a	4.03±0.14a	1.35±0.10b	5.40±1.32a
Leu	5.06±0.02a	4.52±0.23a	1.35±0.10b	6.15±1.52a
Lys	3.99±0.16a	4.14±0.16a	3.82±0.31a	4.57±1.10a
Met	0.07±0.01a	0.01±0.00b	0.04±0.00ab	0.07±0.03a
Phe	7.63±0.41ab	11.29±0.86a	4.92±0.21b	11.36±2.89a
Pro	10.89±1.06a	8.79±1.07ab	5.39±0.44b	11.17±3.67a
Ser	8.12±1.24ab	10.98±0.75a	3.89±0.76b	9.15±2.79a
Tyr	5.71±0.23a	7.07±0.10a	1.63±0.18b	5.79±1.42a
Thr	3.52±0.39b	2.97±0.13b	5.41±0.64a	3.40±0.88b
Val	7.12±0.27a	6.17±0.08a	2.32±0.16b	7.73±1.85a

Values are means±SDs, n=3. Significant differences ($P < 0.05$) among the four N treatments are indicated by different lowercase letters.
