# Supplementary material for: Growth and Physiological Traits of Blueberry Seedlings in Response to Different Nitrogen Forms
Source: Plants (Basel). 2025 May 12;14(10):1444. doi: 10.3390/plants14101444 (PMC12114734; doi:10.3390/plants14101444)
Supplement: Supplementary file 1 [file plants-14-01444-s001.zip › Supplementary tables (anova table).pdf]

Supplementary Table S2 Anova analysis of blueberries' growth indicators and carbon (C) and nitrogen (N) content

Parameters		Sum of squares	Degrees of freedom	Mean square	F-value	P-value
Plant height	Between Groups	2532.667	3	844.222	155.856	0.000
	Within Groups	43.333	8	5.417		
	Total	2576.000	11			
Stem diameter	Between Groups	10.514	3	3.505	54.364	0.000
	Within Groups	0.516	8	0.064		
	Total	11.030	11			
Fresh weight	Between Groups	6994.059	3	2331.353	151.894	0.000
	Within Groups	122.789	8	15.349		
	Total	7116.847	11			
Dry weight	Between Groups	2048.944	3	682.981	95.281	.000
	Within Groups	57.345	8	7.168		
	Total	2106.288	11			
Total C (root)	Between Groups	39.878	3	13.293	36.069	.000
	Within Groups	2.948	8	0.369		
	Total	42.826	11			
Total C (stem)	Between Groups	1.475	3	0.492	4.674	0.036
	Within Groups	0.841	8	0.105		
	Total	2.316	11			
Total C (leaf)	Between Groups	3.215	3	1.072	6.740	0.014
	Within Groups	1.272	8	0.159		
	Total	4.487	11			
Total N (root)	Between Groups	1.919	3	0.640	775.434	0.000
	Within Groups	0.007	8	0.001		
	Total	1.926	11			
Total N (stem)	Between Groups	0.325	3	0.108	227.766	0.000
	Within Groups	0.004	8	0.000		
	Total	0.329	11			
Total N (leaf)	Between Groups	1.185	3	0.395	894.214	0.000
	Within Groups	0.004	8	0.000		
	Total	1.188	11			
C/N ratio (root)	Between Groups	1243.229	3	414.410	263.940	0.000
	Within Groups	12.561	8	1.570		
	Total	1255.790	11			
C/N ratio (stem)	Between Groups	2180.692	3	726.897	306.046	0.000
	Within Groups	19.001	8	2.375		

	Total	2199.693	11			
C/N ratio (leaf)	Between Groups	929.794	3	309.931	1207.539	0.000
	Within Groups	2.053	8	0.257		
	Total	931.847	11			

Supplementary Table S3 Anova analysis of blueberries' photosynthetic parameters and chlorophyll content

Parameters		Sum of squares	Degrees of freedom	Mean square	F-value	P-value
Pn	Between Groups	164.475	3	54.825	175.407	0.000
	Within Groups	2.500	8	0.313		
	Total	166.975	11			
Tr	Between Groups	12.627	3	4.209	31.393	0.000
	Within Groups	1.073	8	0.134		
	Total	13.700	11			
Ci	Between Groups	6874.148	3	2291.383	56.102	0.000
	Within Groups	326.748	8	40.843		
	Total	7200.895	11			
Gs	Between Groups	0.085	3	0.028	73.549	0.000
	Within Groups	0.003	8	0.000		
	Total	0.088	11			
LWUE	Between Groups	3.818	3	1.273	65.061	0.000
	Within Groups	0.156	8	0.020		
	Total	3.974	11			
LUE	Between Groups	0.000	3	0.000	175.407	0.000
	Within Groups	0.000	8	0.000		
	Total	0.000	11			
Chl *a*	Between Groups	0.135	3	0.045	112.531	0.000
	Within Groups	0.003	8	0.000		
	Total	0.139	11			
Chl *b*	Between Groups	0.022	3	0.007	91.059	0.000
	Within Groups	0.001	8	0.000		
	Total	0.022	11			
Chl (*a*+*b*)	Between Groups	0.265	3	0.088	111.855	0.000
	Within Groups	0.006	8	0.001		
	Total	0.271	11			

Supplementary Table S4 Anova analysis of blueberries' antioxidant system indexes, flavonoids, ellagic acid and

sugar contents

Parameters		Sum of squares	Degrees of freedom	Mean square	F-value	P-value
MDA	Between Groups	149.975	3	49.992	1436.525	0.000
	Within Groups	0.278	8	0.035		
	Total	150.254	11			
H_2O_2	Between Groups	117.756	3	39.252	2175.409	0.000
	Within Groups	0.144	8	0.018		
	Total	117.900	11			
$O_2^{\cdot -}$	Between Groups	5.430	3	1.810	75.372	0.000
	Within Groups	0.192	8	0.024		
	Total	5.622	11			
Soluble protein	Between Groups	0.463	3	0.154	12.345	0.002
	Within Groups	0.100	8	0.013		
	Total	0.564	11			
SOD	Between Groups	2663.854	3	887.951	62.224	0.000
	Within Groups	114.163	8	14.270		
	Total	2778.016	11			
POD	Between Groups	4431.463	3	1477.154	36.257	0.000
	Within Groups	325.926	8	40.741		
	Total	4757.389	11			
AsA	Between Groups	101.559	3	33.853	38.945	0.000
	Within Groups	6.954	8	0.869		
	Total	108.513	11			
GSH	Between Groups	6017.086	3	2005.695	493.392	0.000
	Within Groups	32.521	8	4.065		
	Total	6049.607	11			
Flvaonoids	Between Groups	0.106	3	0.035	58.104	0.000
	Within Groups	0.005	8	0.001		
	Total	0.111	11			
Ellagic acid	Between Groups	5.342	3	1.781	70.208	0.000
	Within Groups	0.203	8	0.025		
	Total	5.545	11			
Glucose	Between Groups	9.896	3	3.299	27.994	0.000
	Within Groups	0.943	8	0.118		
	Total	10.839	11			
Fructose	Between Groups	117.804	3	39.268	14.326	0.001

	Within Groups	21.928	8	2.741		
	Total	139.732	11			
Sucrose	Between Groups	3115.031	3	1038.344	161.416	0.000
	Within Groups	51.462	8	6.433		
	Total	3166.493	11			
Total sugar	Between Groups	3676.333	3	1225.388	94.500	0.000
	Within Groups	103.737	8	12.967		
	Total	3779.902	11			

Supplementary Table S5 Anova analysis of blueberries' free amino acid content

Parameters		Sum of squares	Degrees of freedom	Mean square	F-value	P-value
Asp	Between Groups	16.299	3	5.433	25.364	0.000
	Within Groups	1.714	8	0.214		
	Total	18.013	11			
Ala	Between Groups	56.672	3	18.891	17.151	0.001
	Within Groups	8.811	8	1.101		
	Total	65.484	11			
Arg	Between Groups	13379.056	3	4459.685	485.998	0.000
	Within Groups	73.411	8	9.176		
	Total	13452.467	11			
Cys	Between Groups	1.089	3	0.363	19.769	0.000
	Within Groups	0.147	8	0.018		
	Total	1.236	11			
Glu	Between Groups	1.925	3	0.642	2.013	0.191
	Within Groups	2.550	8	0.319		
	Total	4.475	11			
Gly	Between Groups	0.634	3	0.211	25.085	0.000
	Within Groups	0.067	8	0.008		
	Total	0.702	11			
His	Between Groups	218.665	3	72.888	216.683	0.000
	Within Groups	2.691	8	0.336		
	Total	221.356	11			
Ile	Between Groups	3.909	3	1.303	7.246	0.011
	Within Groups	1.439	8	0.180		
	Total	5.348	11			
Leu	Between Groups	7.731	3	2.577	12.090	0.002

	Within Groups	1.705	8	0.213		
	Total	9.436	11			
Lys	Between Groups	37.776	3	12.592	74.380	0.000
	Within Groups	1.354	8	0.169		
	Total	39.130	11			
Met	Between Groups	0.004	3	0.001	28.186	0.000
	Within Groups	0.000	8	0.000		
	Total	0.004	11			
Phe	Between Groups	64.308	3	21.436	25.007	0.000
	Within Groups	6.858	8	0.857		
	Total	71.166	11			
Pro	Between Groups	38.756	3	12.919	8.716	0.000
	Within Groups	11.857	8	1.482		
	Total	50.612	11			
Ser	Between Groups	46.904	3	15.635	13.260	0.002
	Within Groups	9.432	8	1.179		
	Total	56.336	11			
Tyr	Between Groups	18.960	3	6.320	31.489	0.000
	Within Groups	1.606	8	0.201		
	Total	20.565	11			
Thr	Between Groups	110.368	3	36.789	108.196	0.000
	Within Groups	2.720	8	0.340		
	Total	113.088	11			
Val	Between Groups	9.651	3	3.217	10.192	0.004
	Within Groups	2.525	8	0.316		
	Total	12.176	11			
ΣEAA	Between Groups	1611.316	3	537.105	31.571	0.000
	Within Groups	136.102	8	17.013		
	Total	1747.418	11			
ΣNEAA	Between Groups	16877.063	3	5625.688	194.047	0.000
	Within Groups	231.931	8	28.991		
	Total	17108.994	11			
ΣTAA	Between Groups	27872.146	3	9290.715	108.027	0.000
	Within Groups	688.030	8	86.004		
	Total	28560.175	11			

Supplementary Table S6 Anova analysis of blueberries' key enzyme activities involved in the N metabolism

Parameters		Sum of squares	Degrees of freedom	Mean square	F-value	P-value
GDH	Between Groups	46447.931	3	15482.644	115.060	0.000
	Within Groups	1076.490	8	134.561		
	Total	47524.421	11			
GOGAT	Between Groups	1712.118	3	570.706	18.492	0.001
	Within Groups	246.898	8	30.862		
	Total	1959.016	11			
GS	Between Groups	15779.557	3	5259.852	717.566	0.000
	Within Groups	58.641	8	7.330		
	Total	15838.199	11			

Supplementary Table S7 Anova analysis of blueberry cultivation substrates' physicochemical parameters

Parameters		Sum of squares	Degrees of freedom	Mean square	F-value	P-value
pH	Between Groups	4.938	3	1.646	1463.081	0.000
	Within Groups	0.009	8	0.001		
	Total	4.947	11			
EC	Between Groups	8.689	3	2.896	3131.207	0.000
	Within Groups	0.007	8	0.001		
	Total	8.697	11			
Organic matter	Between Groups	86.890	3	28.963	43.804	0.000
	Within Groups	5.290	8	0.661		
	Total	92.180	11			
Organic carbon	Between Groups	29.243	3	9.748	44.088	0.000
	Within Groups	1.769	8	0.221		
	Total	31.012	11			
Alkali-hydrolyzable N	Between Groups	2907209.853	3	969069.951	2885.740	0.000
	Within Groups	2686.507	8	335.813		
	Total	2909896.360	11			
