# Supplementary figures and images for: Growth and Physiological Traits of Blueberry Seedlings in Response to Different Nitrogen Forms
Source: Plants (Basel). 2025 May 12;14(10):1444. doi: 10.3390/plants14101444 (PMC12114734; doi:10.3390/plants14101444)

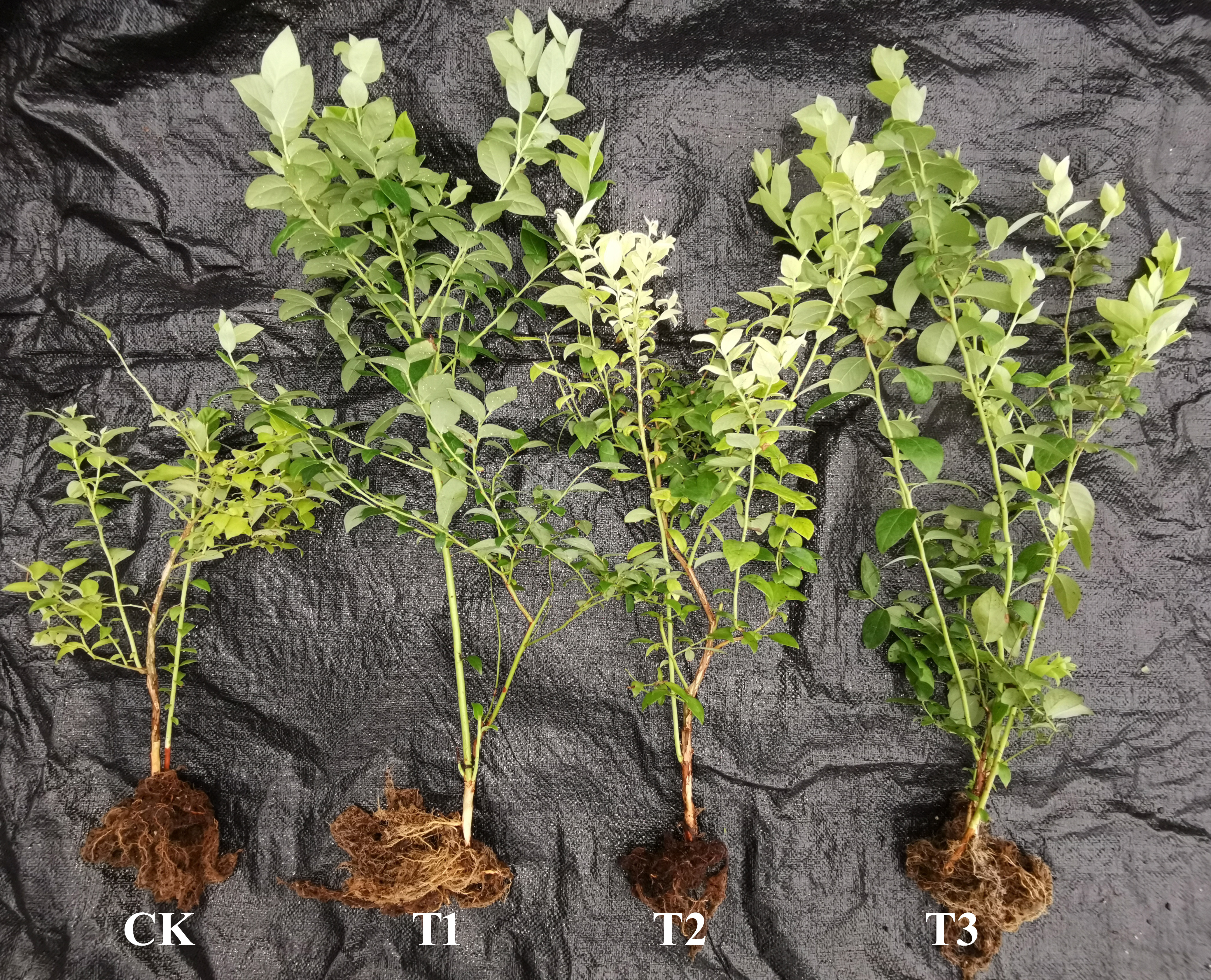

Supplement: Supplementary file 1 [file plants-14-01444-s001.zip › Supplementary Figure S1.jpg]
